# Synthesis of Reactive Sulfur Species in Cultured Vascular Endothelial Cells after Exposure to TGF-β_1_: Induction of Cystathionine γ-Lyase and Cystathionine β-Synthase Expression Mediated by the ALK5-Smad2/3/4 and ALK5-Smad2/3-ATF4 Pathways

**DOI:** 10.3390/ijms222111762

**Published:** 2021-10-29

**Authors:** Musubu Takahashi, Tomoya Fujie, Tsuyoshi Nakano, Takato Hara, Yasuhiro Shinkai, Ryoko Takasawa, Yasushi Hara, Yoshito Kumagai, Chika Yamamoto, Toshiyuki Kaji

**Affiliations:** 1 Faculty of Pharmaceutical Sciences, Tokyo University of Science, 2641 Yamazaki, Noda 278-8510, Japan; 3A19703@ed.tus.ac.jp (M.T.); t-nakano@rs.tus.ac.jp (T.N.); takasawa@rs.tus.ac.jp (R.T.); 2 Faculty of Pharmaceutical Sciences, Toho University, 2-2-1 Miyama, Funabashi 274-8510, Japan; t-fujie@phar.toho-u.ac.jp (T.F.); takato.hara@phar.toho-u.ac.jp (T.H.); 3 Environmental Biology Laboratory, Faculty of Medicine, University of Tsukuba, 1-1-1 Tennodai, Tsukuba 305-8575, Japan; ya_shinkai@md.tsukuba.ac.jp (Y.S.); yk-em-tu@md.tsukuba.ac.jp (Y.K.); 4 Research Institute for Biomedical Sciences, Tokyo University of Science, 2669 Yamazaki, Noda 278-0022, Japan; hara_yasushi@rs.tus.ac.jp

**Keywords:** endothelial cell, cystathionine γ-lyase, cystathionine β-synthase, transforming growth factor-β_1_, reactive sulfur species

## Abstract

Transforming growth factor-β_1_ (TGF-β_1_) occurs at high levels at damage sites of vascular endothelial cell layers and regulates the functions of vascular endothelial cells. Reactive sulfur species (RSS), such as cysteine persulfide, glutathione persulfide, and hydrogen persulfide, are cytoprotective factors against electrophiles such as reactive oxygen species and heavy metals. Previously, we reported that sodium trisulfide, a sulfane sulfur donor, promotes vascular endothelial cell proliferation. The objective of the present study was to clarify the regulation and significance of RSS synthesis in vascular endothelial cells after exposure to TGF-β_1_. Bovine aortic endothelial cells in a culture system were treated with TGF-β_1_ to assess the expression of intracellular RSS, the effect of RSS on cell proliferation in the presence of TGF-β_1_, induction of RSS-producing enzymes by TGF-β_1_, and intracellular signal pathways that mediate this induction. The results suggest that TGF-β_1_ increased intracellular RSS levels to modulate its inhibitory effect on proliferation. The increased production of RSS, probably high-molecular-mass RSS, was due to the induction of cystathionine γ-lyase and cystathionine β-synthase, which are RSS-producing enzymes, and the induction was mediated by the ALK5-Smad2/3/4 and ALK5-Smad2/3-ATF4 pathways in vascular endothelial cells. TGF-β_1_ regulates vascular endothelial cell functions such as proliferation and fibrinolytic activity; intracellular high-molecular-mass RSS, which are increased by TGF-β_1_, may modulate the regulation activity in vascular endothelial cells.

## 1. Introduction

Vascular endothelial cells cover the vascular lumen in a monolayer and function as a barrier between the blood and the subendothelial matrix. The cells regulate the blood coagulation–fibrinolytic system by synthesizing and secreting prostacyclin [1], heparan sulfate [2] and dermatan sulfate [3,4] proteoglycans, and tissue plasminogen activator [5]. Repeated and severe injury of endothelial monolayer initiates the blood coagulation process. Hence, damage to the endothelial monolayer is a causative factor of vascular lesions such as atherosclerosis [6] due to the activated procoagulant state of the blood. As vascular endothelial cells are in direct contact with the blood, their monolayers can be repeatedly or severely injured by toxic environmental and nutritional substances. At the site of damage, platelets adhere and aggregate to release transforming growth factor-β_1_ (TGF-β_1_) largely from α-granules [6]. In the atherosclerotic vascular wall, TGF-β_1_ is also released from vascular smooth muscle cells, macrophages, and T lymphocytes, and as a result, TGF-β_1_ is present at high levels at damage sites of vascular endothelial cell layers [6].

TGF-β_1_ regulates vascular endothelial cell functions to maintain fibrin clots and promote the repair of a damaged vascular wall. This cytokine reduces endothelial fibrinolytic activity [7], inhibits vascular endothelial cell proliferation [8], and regulates endothelial production of extracellular matrix components, including fibronectin and proteoglycans [4,9,10]. TGF-β_1_ likely promotes the repair process after injury by enhancing the procoagulant properties of the blood and stimulating extracellular matrix production. TGF-β_1_ signaling is mediated by the TGF-β receptors ALK1 and ALK5 on the cell membranes of vascular endothelial cells [11]. ALK1 and ALK5 differ with respect to the distribution of expression and downstream effectors. In particular, ALK1 is expressed specifically in vascular endothelial cells and activates Smad1/5/8, whereas ALK5 is expressed ubiquitously and activates Smad2/3. Activated Smads such as Smad1/5/8 and Smad2/3 form a complex with the common partner Smad, i.e., Smad4, and move into the nucleus to induce the transcription of target genes. In addition to the Smad pathway, non-Smad pathways—the mitogen-activated protein kinase (MAPK) pathways, including the extracellular signal-regulated kinase 1/2 (ERK1/2), p38 MAPK, and c-jun N-terminal kinase (JNK) signaling pathways [12,13]―are activated by TGF-β_1_.

Reactive sulfur species (RSS), such as cysteine persulfide, glutathione persulfide, and hydrogen persulfide, can act as cytoprotective factors against electrophiles, including reactive oxygen species (ROS) and heavy metals [14,15,16]. Previously, we found that transcription of the gene encoding cystathionine γ-lyase (CSE), an RSS-producing enzyme, is induced by copper diethyldithiocarbamate through activation of the ERK1/2, p38 MAPK, and hypoxia-inducible factor-1α/β (HIF-1α/β) pathways in vascular endothelial cells [17]. Additionally, we revealed that fibroblast growth factor-2 (FGF-2) increases intracellular RSS levels by inducing CSE expression through the ERK1/2 pathway in vascular endothelial cells [18]. Furthermore, we recently observed that sodium trisulfide, a sulfane sulfur donor, promotes vascular endothelial cell proliferation [19]. These results suggest that RSS modulate the regulation of vascular endothelial cell functions by growth factors/cytokines via the induction of specific RSS-producing enzymes.

We hypothesized that RSS may modulate the regulation of vascular endothelial functions, especially proliferation, by TGF-β_1_. The objectives of the present study were (1) to investigate whether TGF-β_1_ regulates intracellular RSS levels, (2) to test whether RSS can modulate the inhibitory effect of TGF-β_1_ on proliferation, (3) to identify the type(s) of RSS-producing enzymes whose expression is regulated by TGF-β_1_, and (4) to elucidate the intracellular signaling pathway responsible for TGF-β_1_-dependent regulation of expression of RSS-producing enzyme(s) using a culture system of bovine aortic endothelial cells.

## 2. Results

### 2.1. TGF-β_1_ Increases RSS Levels in Vascular Endothelial Cells

**Figure 1:**
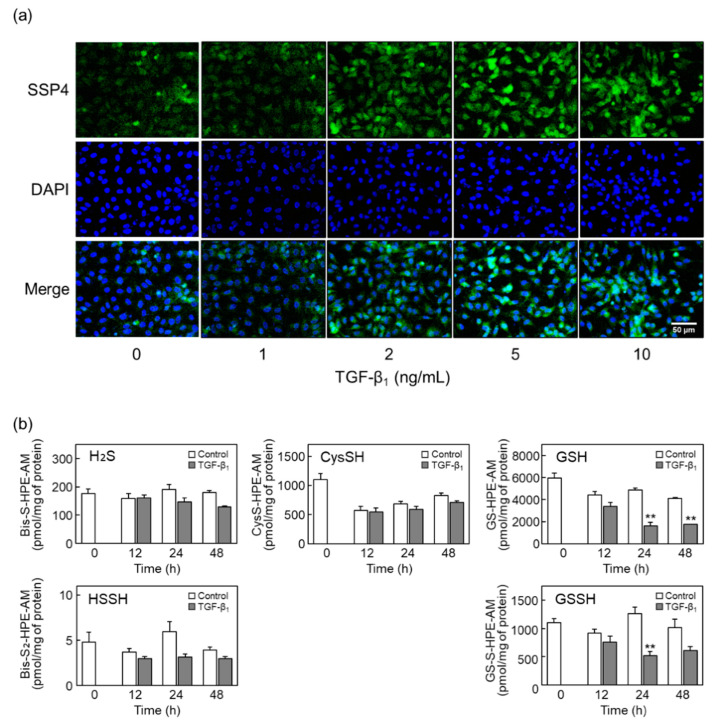
Effects of TGF-β_1_ on intracellular RSS levels in vascular endothelial cells. (**a**) Fluorescence imaging of sulfane sulfur in vascular endothelial cells treated with TGF-β_1_. Confluent cultures of vascular endothelial cells were treated with TGF-β_1_ (1, 2, 5, or 10 ng/mL) for 24 h and were then stained with SSP4 (for sulfane sulfur, green) and DAPI (for nuclei, blue). Scale bar = 50 µm. The data are representative of two independent experiments. (**b**) Quantification of low-molecular-mass RSS and the corresponding substrates in vascular endothelial cells treated with TGF-β_1_. Confluent cultures of vascular endothelial cells were treated with TGF-β_1_ (10 ng/mL) for 12, 24, or 48 h, and the concentration of each molecule was measured using LC-ESI-MS/MS. Values are the mean ± standard error (S.E.) of three independent samples. Data were analyzed using Student’s *t*-test. ** Significantly different from the corresponding control, *p* < 0.01.

First, we examined whether TGF-β_1_ affected RSS levels in vascular endothelial cells (Figure 1). When total sulfane sulfur levels in vascular endothelial cells treated with TGF-β_1_ were detected using SSP4 (a sulfane-sulfur-specific fluorescent probe), intracellular sulfane sulfur levels were increased by TGF-β_1_ in a concentration-dependent manner after 24 h treatments (Figure 1a). Additionally, low-molecular-mass RSS (i.e., hydrogen persulfide, cysteine persulfide, and glutathione persulfide) and the corresponding substrates (i.e., hydrogen sulfide, cysteine, and glutathione) were quantified in vascular endothelial cells treated with TGF-β_1_ (Figure 1b); cysteine persulfide was not detected. TGF-β_1_ did not elevate the levels of any low-molecular-mass RSS (glutathione and glutathione persulfide were significantly decreased), suggesting that TGF-β_1_ increased the levels of high-molecular-mass RSS (i.e., protein persulfide/polysulfide) in vascular endothelial cells.

### 2.2. RSS Modulate TGF-β_1_ Inhibition of Vascular Endothelial Cell Proliferation

**Figure 2:**
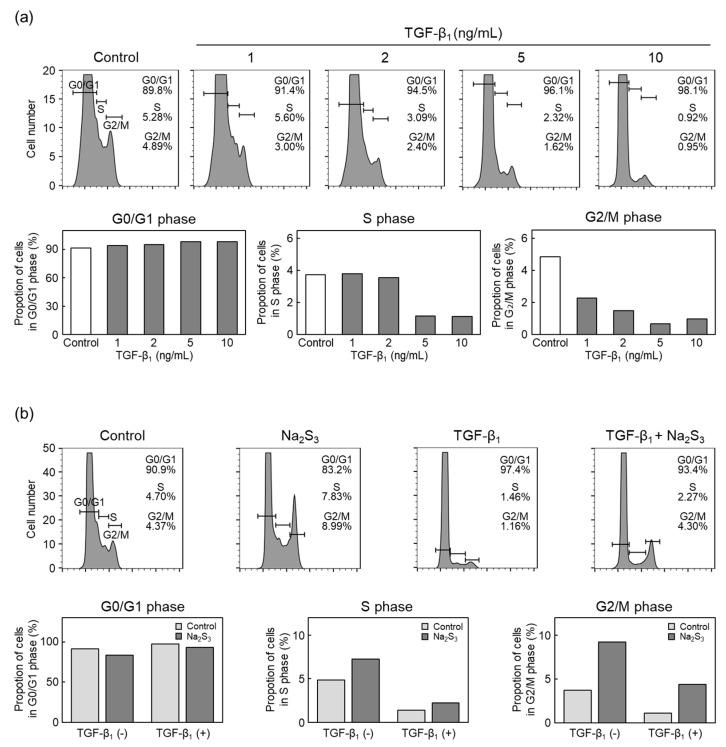
Effects of RSS on TGF-β_1_-induced inhibition of vascular endothelial cell proliferation. (**a**) Cell cycle distribution of vascular endothelial cells treated with TGF-β_1_. Sparse cultures of vascular endothelial cells were treated with TGF-β_1_ (1, 2, 5, or 10 ng/mL) for 24 h and were stained with propidium iodide. DNA histograms of the cell cycle were analyzed (upper panels), and the proportions of cells in G0/G1, S, and G2/M phases were quantified from the histograms (lower panels). The data are representative of two independent experiments. (**b**) Cell cycle distribution of vascular endothelial cells treated with Na_2_S_3_ and TGF-β_1_. Sparse cultures of vascular endothelial cells were pretreated with Na_2_S_3_ (100 µM) for 3 h and then treated with TGF-β_1_ (10 ng/mL) for 24 h. The DNA histograms of the proportions of cells in G0/G1, S, and G2/M phases were quantified (lower panels). The data are representative of three independent experiments.

To investigate whether RSS modulate the inhibition of vascular endothelial cell proliferation by TGF-β_1_ [20], the cell cycle phase of vascular endothelial cells treated with TGF-β_1_ was analyzed using flow cytometry (Figure 2). TGF-β_1_ slightly increased the proportion of cells in the G0/G1 phase and markedly decreased the proportion of cells in the S and G2/M phases (Figure 2a). This result is consistent with a previous report showing that TGF-β_1_ inhibits vascular endothelial cell proliferation by G1 phase arrest [8].

The effects of sodium trisulfide on changes in the cell cycle distribution caused by TGF-β_1_ in vascular endothelial cells were investigated to test whether RSS can modulate the inhibitory effect of TGF-β_1_ on vascular endothelial cell proliferation (Figure 2b). Sodium trisulfide alone increased the proportion of cells in S and G2/M phases, and TGF-β_1_ alone markedly decreased the proportion of cells in these phases. Sodium trisulfide partially recovered the decrease in the proportion of cells in the S and G2/M phases due to TGF-β_1_, suggesting that an increase in intracellular RSS level can modulate inhibitory effects of TGF-β_1_ on vascular endothelial cell proliferation.

### 2.3. TGF-β_1_ Induces the Expression of CSE and Cystathionine β-Synthase (CBS) among RSS-Producing Enzymes

**Figure 3:**
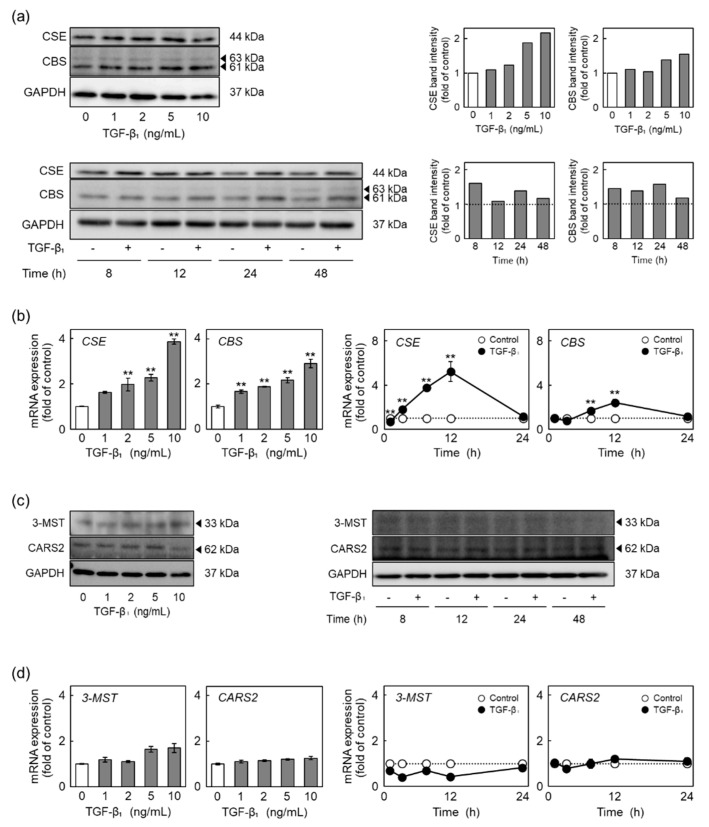
Effects of TGF-β_1_ on the expression of RSS-producing enzymes in vascular endothelial cells. (**a**) Levels of CSE and CBS proteins in vascular endothelial cells treated with TGF-β_1_. Confluent cultures of vascular endothelial cells were treated with TGF-β_1_ (1, 2, 5, or 10 ng/mL) for 24 h (upper panels) or treated with TGF-β_1_ (10 ng/mL) for 8, 12, 24, or 48 h (lower panels). The arrowheads indicate the corresponding protein. Quantification of band intensities of CSE and CBS (right panels). The data are representative of two independent experiments. (**b**) Expression of CSE and CBS mRNAs in vascular endothelial cells treated with TGF-β_1_. Confluent cultures of vascular endothelial cells were treated with TGF-β_1_ (1, 2, 5, or 10 ng/mL) for 12 h (left panels) or treated with TGF-β_1_ (10 ng/mL) for 2, 4, 8, 12, or 24 h (right panels). (**c**) Levels of 3-MST and CARS2 proteins in vascular endothelial cells treated with TGF-β_1_. Confluent cultures of vascular endothelial cells were treated with TGF-β_1_ (1, 2, 5, or 10 ng/mL) for 24 h (left panel) or treated with TGF-β_1_ (10 ng/mL) for 8, 12, 24, or 48 h (right panel). The arrowheads indicate the corresponding protein. The data are representative of two independent experiments. (**d**) Expression of 3-MST and CARS2 mRNAs in vascular endothelial cells treated with TGF-β1. Confluent cultures of vascular endothelial cells were treated with TGF-β_1_ (1, 2, 5, or 10 ng/mL) for 12 h (left panels) or treated with TGF-β_1_ (10 ng/mL) for 2, 4, 8, 12, or 24 h (right panels). Values are the mean ± S.E. of three independent samples in Figure 3b,d. The data were analyzed using one-way ANOVA, followed by Dunnett’s test (Figure 3b,d, left panels) or Student’s *t*-test (Figure 3b,d, right panels). ** Significantly different from the corresponding control, *p* < 0.01.

RSS are produced by CSE [15], CBS [15], 3-mercaptopyruvate sulfurtransferase (3-MST) [21], and cysteinyl-tRNA synthetase 2 (CARS2) [22]. To identify RSS-producing enzyme(s) responsible for the increase in intracellular RSS levels due to TGF-β_1_, the expression levels of RSS-producing enzymes in vascular endothelial cells treated with TGF-β_1_ were determined (Figure 3). TGF-β_1_ increased the levels of CSE and CBS proteins in a concentration-dependent manner (Figure 3a, left panel). The increase in the levels of both CSE and CBS proteins occurred after 8 h and more, respectively (Figure 3a, right panel). The levels of both CSE and CBS mRNAs were also elevated by TGF-β_1_ in a concentration-dependent manner (Figure 3b, left two panels). Higher CSE and CBS mRNA levels were observed after 4 h and more and after 8 h and more, respectively; however, this was not observed after 24 h of TGF-β_1_ treatment (Figure 3b, right two panels). The levels of 3-MST and CARS2 proteins and mRNAs were not affected by TGF-β_1_ (Figure 3c,d). These results indicated that TGF-β_1_ selectively induced CSE and CBS expression in vascular endothelial cells with respect to the four examined RSS-producing enzymes. CSE and CBS are suggested to be responsible for the modulation of the inhibitory effect of TGF-β_1_ on vascular endothelial cell proliferation.

### 2.4. The ALK5-Smad2/3/4 Pathway Mediates Induction of CSE and CBS Expression by TGF-β_1_

**Figure 4:**
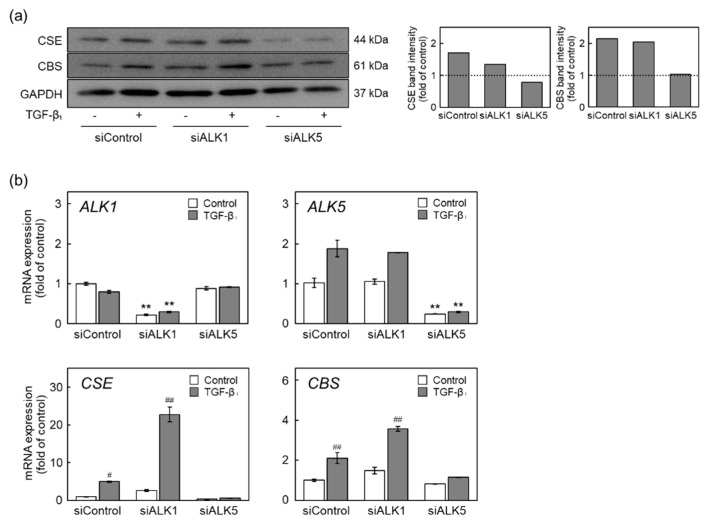
Involvement of ALK1 and ALK5 in the upregulation of CSE and CBS expression by TGF-β_1_ in vascular endothelial cells. (**a**) Levels of CSE and CBS proteins in ALK1- or ALK5-suppressed vascular endothelial cells treated with TGF-β_1_. Subconfluent cultures of vascular endothelial cells were transfected with either ALK1 or ALK5 siRNA for 4 h and were then treated with TGF-β_1_ (10 ng/mL) for 24 h. Quantification of band intensities of CSE and CBS (right panels). The data are representative of two independent experiments. (**b**) Expression of ALK1, ALK5, CSE, and CBS mRNAs in ALK1- or ALK5-suppressed vascular endothelial cells treated with TGF-β_1_. Subconfluent cultures of vascular endothelial cells were transfected with either ALK1 or ALK5 siRNA for 4 h and then treated with TGF-β_1_ (10 ng/mL) for 12 h. Values are means ± S.E. of three independent samples. The data were analyzed using one-way ANOVA, followed by Tukey–Kramer’s test. ** Significantly different from the corresponding siControl, *p* < 0.01. Significantly different from the corresponding control, ^#^ *p* < 0.05; ^##^ *p* < 0.01.

We investigated the signaling pathways that mediated the induction of CSE and CBS expression by TGF-β_1_ in vascular endothelial cells; specifically, the involvement of ALK1, ALK5, TGF-β_1_ type I receptors in the induction of CSE and CBS expression by TGF-β_1_ was examined (Figure 4). Cells were transfected with either small interfering (si)ALK1 or siALK5 and were then treated with TGF-β_1_. TGF-β_1_ significantly elevated the levels of CSE and CBS proteins following ALK1 knockdown and treatment with siControl, whereas TGF-β_1_-mediated elevation was reduced by ALK5 knockdown (Figure 4a). Similarly, induction of CSE and CBS mRNA expression by TGF-β_1_ was abrogated by siALK5 but not by siALK1 (Figure 4b). These results suggest that the induction of CSE and CBS expression by TGF-β_1_ is mediated by ALK5 but not by ALK1. Interestingly, siRNA-mediated knockdown of ALK1 enhanced the induction of CSE and CBS mRNA expression in vascular endothelial cells. This may be due to an increase in TGF-β_1_ bound to ALK5.

**Figure 5:**
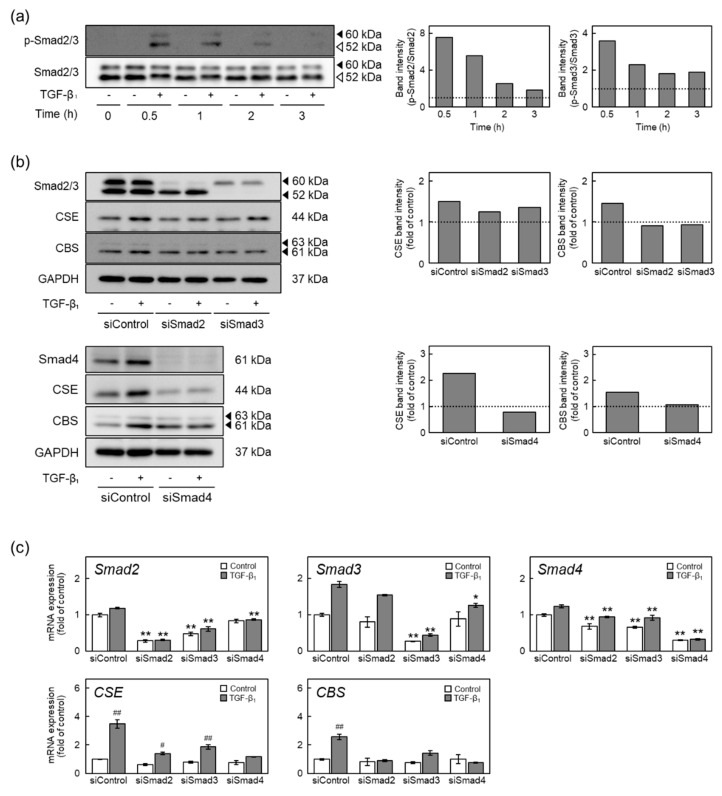
Involvement of Smad2/3/4 in the upregulation of CSE and CBS expression by TGF-β_1_ in vascular endothelial cells. (**a**) Phosphorylation of Smad2/3 in vascular endothelial cells treated with TGF-β_1_. Confluent cultures of vascular endothelial cells were treated with TGF-β_1_ (10 ng/mL) for 0.5, 1, 2, or 3 h. The arrowheads indicate Smad2 protein (solid arrowheads) and Smad3 protein (open arrowheads). Quantification of band intensities of phospho-Smad2/3 and Smad2/3 (right panels). The data are representative of two independent experiments. (**b**) Levels of Smad2, Smad3, Smad4, CSE, and CBS proteins in Smad2-, Smad3-, or Smad4-suppressed vascular endothelial cells treated with TGF-β_1_. Subconfluent cultures of vascular endothelial cells were transfected with either Smad2, Smad3, or Smad4 siRNA for 4 h and then treated with TGF-β_1_ (10 ng/mL) for 24 h. The arrowheads indicate the corresponding protein. Quantification of band intensities of CSE and CBS (right panels). The data are representative of two independent experiments. (**c**) Expression of Smad2, Smad3, Smad4, CSE, and CBS mRNAs in Smad2-, Smad3-, or Smad4-suppressed vascular endothelial cells treated with TGF-β_1_. Subconfluent cultures of vascular endothelial cells were transfected with either Smad2, Smad3, or Smad4 siRNA for 4 h and then treated with TGF-β_1_ (10 ng/mL) for 12 h. Values are means ± S.E. of three independent samples. The data were analyzed using one-way ANOVA, followed by Tukey–Kramer’s test. Significantly different from the corresponding siControl, * *p* < 0.05; ** *p* < 0.01. Significantly different from the corresponding control, ^#^ *p* < 0.05; ^##^ *p* < 0.01.

Because TGF-β_1_-activated ALK5 phosphorylates Smad2 and Smad3 [11], we examined the involvement of Smad2/3 in the induction of CSE and CBS expression by TGF-β_1_ (Figure 5). Phosphorylation of Smad2/3 was immediately increased by TGF-β_1_ at 0.5 h and was maintained for 3 h of TGF-β_1_ treatment (Figure 5a). Induction of CSE protein expression by TGF-β_1_ was abrogated by siSmad4; siSmad2 and siSmad3 weakly reduced the induction. By contrast, induction of CBS protein expression by TGF-β_1_ was abrogated by siSmad2, siSmad3, and siSmad4 (Figure 5b). Additionally, TGF-β_1_-induced elevation of CSE and CBS mRNA expression was reduced by either siSmad2, siSmad3, or siSmad4 (Figure 5c). These results suggest that TGF-β_1_ induces CSE and CBS expression through the ALK5-Smad2/3/4 pathway in vascular endothelial cells.

### 2.5. The ALK5-Smad2/3-ATF4 Pathway Mediates Induction of CSE and CBS Expression by TGF-β_1_

**Figure 6:**
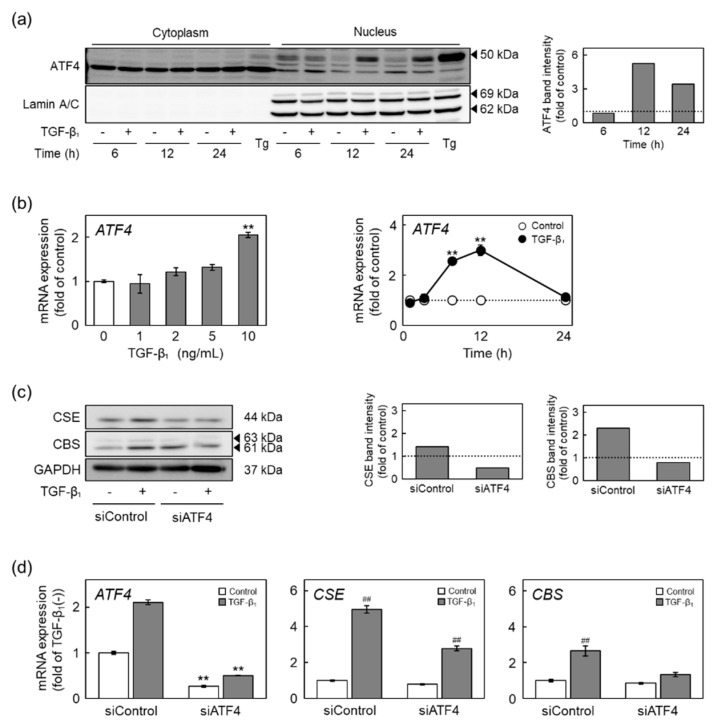
Involvement of ATF4 in the upregulation of CSE and CBS expression by TGF-β_1_ in vascular endothelial cells. (**a**) Levels of ATF4 protein in the cytoplasm and nucleus of vascular endothelial cells treated with TGF-β_1_. Confluent cultures of vascular endothelial cells were treated with TGF-β_1_ (10 ng/mL) for 6, 12, or 24 h or with thapsigargin (Tg; 0.1 µM) as a positive control for 9 h to express ATF4. Lamin A/C are used as a nuclear loading control. The arrowheads indicate the corresponding protein. Quantification of band intensities of ATF4 in nucleus (right panels). The data are representative of three independent experiments. (**b**) Expression of ATF4 mRNA in vascular endothelial cells treated with TGF-β_1_. Confluent cultures of vascular endothelial cells were treated with TGF-β_1_ (1, 2, 5, and 10 ng/mL) for 12 h (left panel) or treated with TGF-β_1_ (10 ng/mL) for 2, 4, 8, 12, or 24 h (right panel). Values are the mean ± S.E. of three independent samples. The data were analyzed using one-way ANOVA, followed by Dunnett’s test (left panel) or Student’s *t*-test (right panel). ** Significantly different from the corresponding control, *p* < 0.01. (**c**) Levels of CSE and CBS proteins in ATF4-suppressed vascular endothelial cells treated with TGF-β_1_. Subconfluent cultures of vascular endothelial cells were transfected with ATF4 siRNA for 4 h and then treated with TGF-β_1_ (10 ng/mL) for 24 h. The arrowheads indicate the corresponding protein. Quantification of band intensities of CSE and CBS (right panels). The data are representative of two independent experiments. (**d**) Expression of ATF4, CSE, and CBS mRNAs in ATF4-suppressed vascular endothelial cells treated with TGF-β_1_. Subconfluent cultures of vascular endothelial cells were transfected with ATF4 siRNA for 4 h and then treated with TGF-β_1_ (10 ng/mL) for 12 h. Values are means ± S.E. of three independent samples. The data were analyzed using one-way ANOVA, followed by Tukey–Kramer’s test. ** Significantly different from the corresponding siControl, *p* < 0.01. ^##^ Significantly different from the corresponding control, *p* < 0.01.

Activating transcription factor 4 (ATF4), a transcription factor induced by amino acid deficiency or hypoxia, was previously found to be involved in the promotion of CSE gene transcription [23,24]. Therefore, we investigated the involvement of ATF4 in TGF-β_1_-mediated induction of CSE and CBS expression in vascular endothelial cells (Figure 6). TGF-β_1_ promoted the translocation of ATF4 from the cytoplasm into the nucleus after 12 h and more (Figure 6a). ATF4 mRNA levels were significantly increased by TGF-β_1_ in a concentration- and time-dependent manner (Figure 6b). TGF-β_1_-induced upregulation of the levels of CSE and CBS proteins (Figure 6c) and mRNAs (Figure 6d) was reduced by siRNA-mediated knockdown of ATF4. These results suggest that translocation of ATF4 into the nucleus and induction of ATF4 by TGF-β_1_ are involved in the induction of CSE and CBS expression by TGF-β_1_ in vascular endothelial cells.

**Figure 7:**
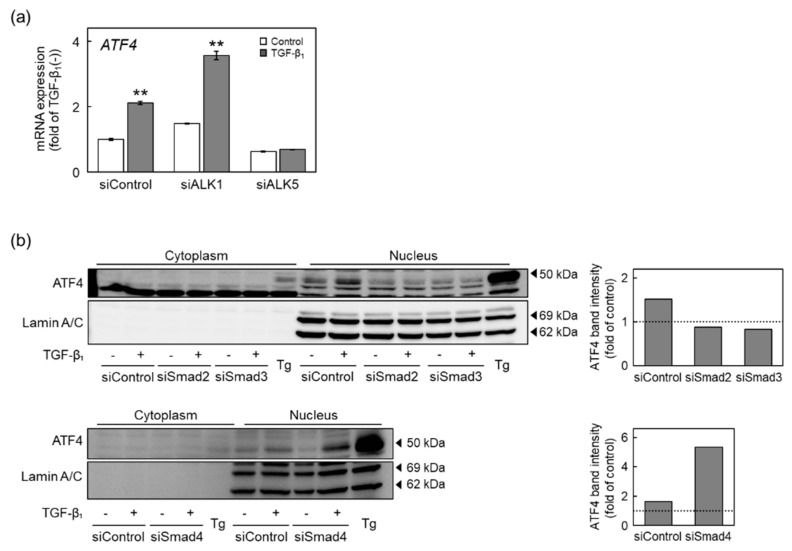
Involvement of the Smad pathway in the upregulation of ATF4 expression by TGF-β_1_ in vascular endothelial cells. (**a**) Expression of ATF4 mRNA in ALK1- or ALK5-suppressed vascular endothelial cells treated with TGF-β_1_. Subconfluent cultures of vascular endothelial cells were transfected with ALK1 or ALK5 siRNA for 4 h and then treated with TGF-β_1_ (10 ng/mL) for 12 h. Values are means ± S.E. of three independent samples. The data were analyzed using one-way ANOVA, followed by Tukey–Kramer’s test. ** Significantly different from the corresponding control, *p* < 0.01. (**b**) Level of ATF4 protein in Smad2-, Smad3-, or Smad4-suppressed vascular endothelial cells treated with TGF-β_1_. Subconfluent cultures of vascular endothelial cells were transfected with either Smad2, Smad3, or Smad4 siRNA for 4 h and then treated with TGF-β_1_ (10 ng/mL) for 12 h or with thapsigargin (Tg; 0.1 µM) as a positive control for 9 h to express ATF4. Lamin A/C are used as a nuclear loading control. The arrowheads indicate the corresponding protein. Quantification of band intensities of ATF4 in nucleus (right panels). The data are representative of two independent experiments.

We analyzed signal transduction of ATF4-containing pathways that mediates the induction of CSE and CBS expression by TGF-β_1_ in vascular endothelial cells (Figure 7). The increase in ATF4 mRNA levels due to TGF-β_1_ was diminished by ALK5 knockdown but not by ALK1 knockdown (Figure 7a), indicating that ATF4 may be a downstream effector of ALK5. Translocation of ATF4 into the nucleus was promoted by TGF-β_1_, and this promotion was diminished by either siSmad2 or siSmad3; however, siSmad4 did not affect the translocation (Figure 7b).

Taken together, these results suggest that the ALK5-Smad2/3 pathway promotes the translocation of ATF4 into the nucleus and induces the expression of CSE and CBS in vascular endothelial cells after exposure to TGF-β_1_, which is independent of Smad4. Thus, TGF-β_1_ induces CSE and CBS expression through the ALK5-Smad2/3-ATF4 pathway without Smad4 in vascular endothelial cells.

### 2.6. The Non-Smad Pathway Does Not Mediate Induction of CSE and CBS Expression by TGF-β_1_

**Figure 8:**
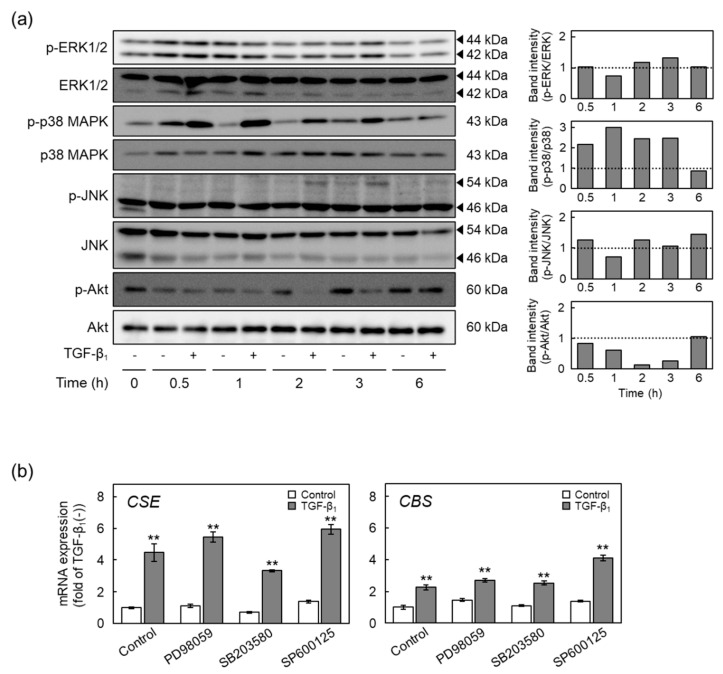
Involvement of the non-Smad pathway in the upregulation of CSE and CBS expression by TGF-β_1_ in vascular endothelial cells. (**a**) Phosphorylation of ERK1/2, p38 MAPK, JNK, and Akt in vascular endothelial cells treated with TGF-β_1_. Confluent cultures of vascular endothelial cells were treated with TGF-β_1_ (10 ng/mL) for 0.5, 1, 2, 3, or 6 h. The arrowheads indicate the corresponding protein. Quantification of band intensities of phospho-ERK1/2, phospho-p38 MAPK, phospho-JNK, and phospho-Akt (right panels). The data are representative of two independent experiments. (**b**) Expression of CSE and CBS mRNAs in vascular endothelial cells pretreated with either ERK1/2, p38 MAPK, or JNK pathway inhibitor and treated with TGF-β_1_. Confluent cultures of vascular endothelial cells were pretreated with either PD98059 (an ERK1/2 pathway inhibitor), SB203580 (a p38 MAPK pathway inhibitor), or SP600125 (a JNK pathway inhibitor) at 10 µM for 1 h and then treated with TGF-β_1_ (10 ng/mL) for 12 h. Values are means ± S.E. of three independent samples. The data were analyzed using one-way ANOVA, followed by Tukey–Kramer’s test. ** Significantly different from the corresponding control, *p* < 0.01.

TGF-β_1_ activates non-Smad pathways, such as the ERK1/2, p38 MAPK, JNK, and Akt pathways [12,13], as well as Smad pathways. The involvement of non-Smad pathways in the induction of CSE and CBS expression by TGF-β_1_ was thus determined (Figure 8). Phosphorylation of ERK1/2, p38 MAPK, and JNK was increased by TGF-β_1_ after 3, 0.5, and 2 h, respectively, and more; however, phosphorylation of Akt was decreased by TGF-β_1_ (Figure 8a). Neither an ERK1/2 pathway inhibitor PD98059, a p38 MAPK inhibitor SB203580, or a JNK inhibitor SP600125 reduced the levels of CSE and CBS mRNAs (Figure 8b). These results suggest that non-Smad pathways are not involved in the induction of CSE and CBS expression by TGF-β_1_ in vascular endothelial cells.

## 3. Discussion

**Figure 9:**
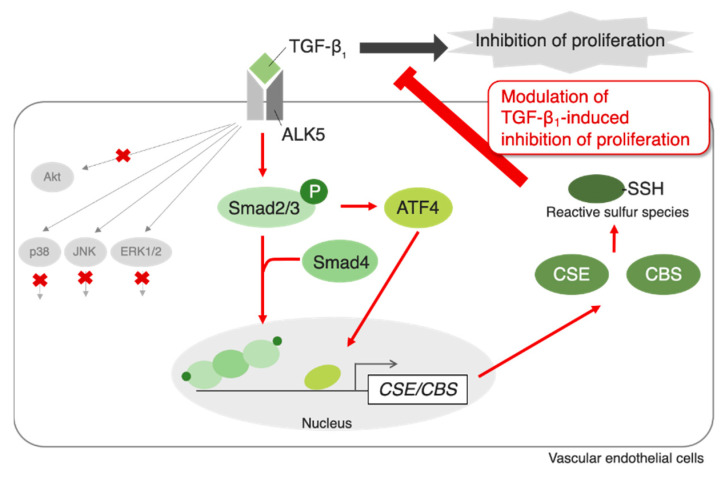
Summary of the present study. TGF-β_1_ activates the ALK5-Smad2/3/4 and ALK5-Smad2/3-ATF4 pathways to induce CSE and CBS, reactive sulfur species-producing enzymes in vascular endothelial cells. The Akt, p38 MAPK, JNK, and ERK1/2 pathways are not involved in this induction. As a result, RSS, particularly high-molecular-mass RSS, are increased. The increased RSS may modulate the regulation by TGF-β_1_ of vascular endothelial cell functions such as inhibition of the proliferation in the repair process of damaged endothelium.

Numerous studies have shown the physiological effects of persulfation/polysulfation on the activity of specific proteins such as calmodulin-dependent protein kinases [25], PTEN [26], and eNOS [27,28]. However, little is known about the functions of RSS in vascular endothelial cells. The role of RSS in TGF-β_1_-induced inhibition of vascular endothelial cell proliferation and regulation of RSS-producing enzymes by TGF-β_1_ was investigated in the present study. The results suggest that TGF-β_1_ increases intracellular RSS levels to modulate its inhibitory effect on proliferation, and the increased production of RSS is likely due to the induction of CSE and CBS expression in vascular endothelial cells. Additionally, the ALK5-Smad2/3/4 and ALK5-Smad2/3-ATF4 pathways mediated the upregulation of CSE and CBS expression to increase intracellular RSS levels in vascular endothelial cells. Although we could not identify the chemical form(s) of the RSS in question, we suggest that the levels of particularly high-molecular-mass RSS are increased by TGF-β_1_. However, sodium tetrasulfide, which is also a sulfane sulfur donor, was reported to persulfidate proteins within the cell; although the most common function of the target proteins was “transcription”, a wide range of other proteins were persulfidated by sodium tetrasulfide [29,30,31,32,33]. Additionally, platelet-derived growth factor (PDGF) increased cellular sulfane sulfur, and tetrasulfide inhibited vascular smooth muscle cell migration through the Akt pathway activated by PDGF-induced ROS production [34], suggesting that there are cell type-dependent systems that regulate RSS production. Thus, TGF-β_1_ likely increases the persulfidation of cellular proteins that positively regulate vascular endothelial cell proliferation to modulate the inhibitory effect of TGF-β_1_ on proliferation. The results of this study are summarized in Figure 9.

In the current study, vascular endothelial cells expressed CSE, CBS, 3-MST, and CARS2 as RSS-producing enzymes. The present results suggest that TGF-β_1_ markedly induced CSE expression and moderately induced CBS expression and that the expression of 3-MST and CARS2 was not affected by TGF-β_1_. Recently, we reported that among RSS-producing enzymes, the expression of CSE is selectively induced by FGF-2 [19]. Additionally, copper diethyldithiocarbamate (Cu10), a copper complex, also selectively enhances the transcriptional induction of CSE without changing the levels of other RSS-producing enzymes [17]. These results suggest that intracellular RSS levels are regulated particularly by the activity of CSE, and other RSS-producing enzymes such as CBS, 3-MST, and CARS2 contribute to the constitutive levels of RSS in vascular endothelial cells. In fact, there are many reports on factors that induce CSE expression, such as hypoxia [23], protein restriction [24], oxidized low-density lipoprotein [35], and 17β-estradiol [36]; however, further studies are needed to examine the exact physiological role of each RSS-producing enzyme. In the present study, TGF-β_1_ induced the expression of CBS and CSE, suggesting that the regulation of vascular endothelial cell functions through TGF-β_1_ (such as inhibition of proliferation) may require strong modulation by RSS. When vascular endothelial cells are damaged, FGF-2 is leaked from the damaged cells and stimulates the cells near the damaged site. On the other hand, platelets aggregate at the damaged site and release TGF-β_1_. These suggest that vascular endothelial cells near the damaged site are exposed to FGF-2 and TGF-β_1_. Therefore, regulation of RSS synthesis by FGF-2 and TGF-β_1_ may be important in the repair process of damaged endothelium.

In the present study, we analyzed the intracellular signaling pathways that mediate the induction of endothelial CSE and CBS expression by TGF-β_1_ and observed that the induction was mediated by both the ALK5-Smad2/3/4 and ALK5-Smad2/3-ATF4 pathways but not by the ERK1/2, p38 MAPK, JNK, and Akt pathways. FGF-2 induces endothelial CSE expression through the ERK1/2 pathway [19]. By contrast, hypoxia induces CSE expression through the HIF-1α-ATF4 pathway [23]. Edoxaban, an anticoagulant drug, induces CSE and CBS expression by activating the PI3K/Akt pathway [37]. Induction of CSE expression by a calcium-sensing receptor agonist is mediated by the calmodulin signaling pathway [38]. We previously found that transcriptional induction of CSE by Cu10 was mediated by the ERK1/2, p38 MAPK, and HIF-1α/β pathways in vascular endothelial cells [17]. Moreover, we observed that heavy metals can induce endothelial CSE expression through pathways other than those mentioned above (unpublished data). These results suggest that the modulation of vascular endothelial cell functions by RSS involves the induction of CSE expression, and possibly CBS expression, mediated by stimulant-dependent intracellular signaling pathways.

In summary, the results of the present study suggest that (1) TGF-β_1_ increases the levels of intracellular RSS, probably protein persulfide/polysulfide; (2) RSS modulate the inhibitory effect of TGF-β_1_ on proliferation; (3) expression of CSE and CBS is induced by TGF-β_1_ and may be responsible for the increase in intracellular RSS levels; and (4) the induction of CSE and CBS expression by TGF-β_1_ is mediated by both the ALK5-Smad2/3/4 and ALK5-Smad2/3-ATF4 pathways in vascular endothelial cells. By contrast, it has been reported that TGF-β_1_ downregulates CSE expression in human alveolar epithelial cells [39], human breast cancer cells [40], and human kidney epithelial HK-2 cells [41,42]. Vascular endothelial cells may thus be a unique cell type in which TGF-β_1_ induces the expression of CSE and CBS. TGF-β_1_ can activate two signaling pathways (i.e., the ALK1-Smad1/5/8 and ALK5-Smad2/3 pathways), which mediate opposite effects in vascular endothelial cells. For example, vascular endothelial cell proliferation is inhibited by activation of the ALK5-Smad2/3 pathway, whereas the ALK1-Smad1/5/8 pathway mediates cell proliferation [11]. Induction of CSE and CBS expression by TGF-β_1_ was enhanced in ALK1-suppressed endothelial cells, suggesting that the ALK1 signaling pathway activated by TGF-β_1_ may suppress CSE and CBS expression, as observed in other cell types. ALK1-mediated downregulation of CSE and CBS expression may be weaker than their ALK5-mediated upregulation. The ALK5-Smad2/3 pathway mediates the regulation of vascular endothelial cell functions other than proliferation as follows: reduction of fibrinolytic activity through the induction of plasminogen activator inhibitor type 1 expression [11], cell cycle arrest in the G1 phase [43], promotion of apoptosis [44], stimulation of synthesis of perlecan, a large heparan sulfate proteoglycan, biglycan, a small leucine-rich dermatan sulfate proteoglycan [10], syndecan-4, a small transmembrane heparan sulfate proteoglycan [45,46], increase in ROS production [47], and nitric oxide production via the induction of endothelial nitric oxide synthase expression [48]. TGF-β_1_ regulates vascular endothelial cell functions; intracellular high-molecular-mass RSS levels, which are increased by TGF-β_1_, may thus modulate the regulation activity in vascular endothelial cells.

## 4. Materials and Methods

### 4.1. Cell Culture and Treatments

Bovine aortic endothelial cells were obtained from Cell Applications (San Diego, CA, USA) and cultured in Dulbecco’s modified Eagle’s medium (DMEM; Nissui Pharmaceutical, Tokyo, Japan) supplemented with 4 mM L-glutamine (Nacalai Tesque, Kyoto, Japan), 0.5% penicillin–streptomycin mixed solution (Nacalai Tesque), and 10% heat-inactivated fetal bovine serum (FBS, Thermo Fisher Scientific, Waltham, MA, USA) in a humidified atmosphere of 5% CO_2_ at 37 °C. After culturing until confluence, vascular endothelial cells were washed twice with serum-free DMEM and were treated with human recombinant TGF-β_1_ (PeproTech, Cranbury, NJ, USA) diluted in 10 mM citric acid or inhibitor in serum-free DMEM. PD98059 (ERK1/2 pathway inhibitor), SB203580 (p38 MAPK pathway inhibitor), and SP600125 (JNK pathway inhibitor) were obtained from Cayman Chemical (Ann Arbor, MI, USA).

### 4.2. Measurement of Intracellular RSS Levels

Vascular endothelial cells were cultured on glass-bottom dishes until confluence and were treated with TGF-β_1_ for 24 h. After washing with Ca^2+^- and Mg^2+^-free phosphate-buffered saline (CMF-PBS, Nissui Pharmaceutical), vascular endothelial cells were fixed using 4% paraformaldehyde in phosphate-buffered solution (Nacalai Tesque) at room temperature for 25 min. After washing twice with CMF-PBS, vascular endothelial cells were stained with fluorescent working solution containing 20 µmol/L SSP4 (Dojindo, Kumamoto, Japan) and 0.5 mmol/L cetyltrimethylammonium bromide (Nacalai Tesque) in serum-free DMEM at 37 °C for 1 h. After washing with CMF-PBS, serum- and phenol red-free DMEM and Fluoro-KEEPER Antifade Reagent (Nacalai Tesque) were added. Fluorescence images were captured using a BZ-9000 fluorescence microscope (Keyence, Osaka, Japan) at λex = 482 nm and λem = 515 nm to measure the content of sulfane sulfur as an indicator of RSS. Intracellular low-molecular-mass RSS were quantified as described previously [49] using liquid chromatography–electrospray ionization–tandem mass spectrometry (LC-ESI-MS/MS) with β-(4-hydroxyphenyl)ethyl iodoacetamide (Molecular Biosciences, Boulder, CO, USA).

### 4.3. Cell Cycle Analyses

Vascular endothelial cells were seeded at a density of 1.0 × 10^4^ cells/cm^2^ and were cultured for 24 h, followed by treatment with TGF-β_1_ for 24 h in serum-free DMEM. After washing twice with CMF-PBS, vascular endothelial cells were collected using 0.25% trypsin (Thermo Fisher Scientific) and 0.02% ethylene diamine tetraacetic acid (Nacalai Tesque) solution. The collected vascular endothelial cells were centrifuged at 4 °C and 100× *g* for 2 min. The supernatant was removed, and the precipitate was resuspended with CMF-PBS, followed by centrifugation at 4 °C and 100× *g* for 2 min. After the supernatant was removed, vascular endothelial cells were fixed using 70% ethanol (Nacalai Tesque) for 30 min on ice. The suspension was centrifuged at 4 °C and 400× *g* for 5 min, and the supernatant was removed. CMF-PBS and RNAse A solution (Sigma Aldrich, St. Louis, MO, USA) in 50 mM Tris-HCl buffer solution containing 50% glycerol was added, followed by incubation at 37 °C for 20 min. The suspension was centrifuged at 4 °C and 400× *g* for 5 min, and the supernatant was removed. After the precipitate was resuspended with CMF-PBS, DNA was stained with propidium iodide (50 µg/mL; Becton Dickinson, San Jose, CA, USA). The cell cycle phase in vascular endothelial cells was analyzed using flow cytometry (FACSCalibur, Becton Dickinson) and FlowJo software (Becton Dickinson).

### 4.4. siRNA Transfection

To prepare siRNA transfection reagents, siRNA and Lipofectamine RNAiMAX (Thermo Fisher Scientific) were added to Opti-MEM (Thermo Fisher Scientific). The mixture was incubated at room temperature for 5 min, followed by mixing and incubation at room temperature for 20 min. Subconfluent cultures of vascular endothelial cells were washed with penicillin- and streptomycin-free DMEM supplemented with 10% FBS and were incubated with transfection reagent containing 18 nM siRNA and 0.09% Lipofectamine RNAiMAX in penicillin- and streptomycin-free DMEM supplemented with 10% FBS at 37 °C for 4 h. The medium was replaced with penicillin- and streptomycin-free DMEM supplemented with 10% FBS, followed by incubation at 37 °C for 20 h and treatment with TGF-β_1_. Sequences of siRNA strands are summarized in Table 1. siRNAs were obtained from Greiner Bio-One GmbH (Kremsmünster, Austria).

### 4.5. Western Blot Analysis

After incubation, the cell layer was washed twice with cold CMF-PBS. To analyze protein levels in cells, the cell layer was lysed with sodium dodecyl sulfate (SDS) sample buffer containing 2% SDS and 10% glycerol in 50 mM Tris-HCl buffer. The cell lysates were incubated at 95 °C for 10 min. To analyze nuclear translocation of ATF4, the cell layer was harvested with CMF-PBS and was centrifuged at 4 °C and 3000× *g* for 5 min. After removing the supernatant, the cytoplasmic and nuclear fractions were separated using NE-PER Nuclear and Cytoplasmic Extraction Reagents (Thermo Fisher Scientific). Protein concentrations were determined using the Protein Assay BCA kit (Nacalai Tesque), and the samples were incubated with 2-mercaptoethanol and bromophenol blue at 95 °C for 3 min. Proteins were separated using SDS-polyacrylamide gel electrophoresis, followed by transfer from the gel to an Immobilon-P Transfer Membrane (Merck Millipore, Billerica, MA, USA). The membrane was blocked with 5% skim milk or 2% bovine serum albumin in Tris-buffered saline with Tween 20 (TBST; 20 mM Tris-HCl, 15 mM NaCl, and 0.1% Tween 20) at room temperature for 1 h. The membrane was then washed with TBST and incubated with a primary antibody in TBST or XL-Enhancer (Integrale, Tokyo, Japan) at 4 °C overnight. After washing with TBST, the membrane was incubated with HRP-conjugated secondary antibody in TBST or XL-Enhancer at room temperature for 1 h. Immunoreactive bands were visualized using Chemi-Lumi One L (Nacalai Tesque) and were recorded using a LAS-3000 device (Fujifilm, Tokyo, Japan). Anti-CSE rabbit polyclonal antibody was prepared as previously described [16]; anti-CBS mouse monoclonal antibody (M01) was obtained from Abnova Corporation (Taipei, Taiwan); anti-phospho-p44/42 MAPK (ERK1/2; #9101) rabbit polyclonal antibody, anti-p44/42 MAPK (#9102) rabbit polyclonal antibody, anti-phospho-p38 MAPK (#9211) rabbit polyclonal antibody, anti-p38 MAPK (#9212) rabbit polyclonal antibody, anti-phospho-SAPK/JNK (#9255) mouse monoclonal antibody, anti-SAPK/JNK (#9252) rabbit polyclonal antibody, anti-phospho-Akt (D9E) rabbit monoclonal antibody, anti-Akt (C67E7) rabbit monoclonal antibody, anti-phospho-Smad2/3 (#8828) rabbit monoclonal antibody, anti-Smad2/3 (#8685) rabbit monoclonal antibody, and HRP-linked anti-rabbit IgG (#7074) and anti-mouse IgG (#7076) secondary antibodies were obtained from Cell Signaling Technology (Danvers, MA, USA). Anti-GAPDH (#5A12) monoclonal antibody was obtained from Fujifilm Wako Pure Chemical. Anti-MPST (3-MST; D-8) mouse monoclonal antibody, anti-Smad4 (B-8) mouse monoclonal antibody, anti-CREB2 (ATF4; C-20) rabbit polyclonal antibody, and anti-lamin A/C (636) mouse monoclonal antibody were obtained from Santa Cruz Biotechnology (Santa Cruz, CA, USA). Anti-CARS2 (ARP68165_P050) primary antibody was obtained from Aviva Systems Biology (San Diego, CA, USA). Band intensity was quantified using ImageJ software (National Institute of Health, Bethesda, MD, USA).

### 4.6. Real-Time Reverse Transcription-Polymerase Chain Reaction (RT-PCR) Analysis

The cell layer was lysed with QIAzol lysis reagent (Qiagen, Venlo, The Netherlands). Chloroform was added, followed by mixing and centrifugation at 4 °C and 12,000× *g* for 15 min. The supernatants were transferred to a new tube, 70% ethanol was added, and the mixture was centrifuged at 4 °C and 15,000× *g* for 10 min. After centrifugation, the RNA pellet was washed twice with 70% ethanol. The RNA pellet was then air-dried and eluted in RNase-free water. The concentration of total RNA was measured using a NanoDrop spectrophotometer (Thermo Fisher Scientific) followed by dilution to a standard concentration. Complementary DNA (cDNA) was synthesized using the High-Capacity cDNA Reverse Transcription Kit (Applied Biosystems, Foster, CA, USA). Real-time RT-PCR was performed using GeneAce SYBR qPCR mix α (Nippon Gene, Tokyo, Japan), containing 10 ng cDNA and 0.2 µM sense/antisense primers, in a StepOnePlus Real-Time PCR System (Thermo Fisher Scientific). Primers (Table 2) were obtained from Eurofins Genomics (Tokyo, Japan). CSE, CBS, 3-MST, CARS2, ALK1, ALK5, Smad2, Smad3, Smad4, ATF4, and β_2_-microglobulin (B2M) mRNA levels in the samples were quantified using the relative standard curve method.

### 4.7. Statistical Analyses

The data were statistically analyzed using Student’s *t*-test, Tukey–Kramer’s test, or Dunnett’s test implemented in Statcel4 software (OMS Publishing Inc., Tokyo, Japan). Differences were considered significant at *p* < 0.05.

## Figures and Tables

**Table 1:** Sequences of siRNA sense and antisense strands.

Gene	Sense Strand (5′→3′)	Antisense Strand (5′→3′)
ALK1	UUCAUGUCCUCAAAGCUGGGG	CCAGCUUUUGAGGACAUGAAtt
ALK5	UUCAUUUGGCACUCGAUGGUG	CCAUCGAGUGCCAAAUGAAtt
Smad2	UUCAAAACCCUGAUUAACGtt	CGUUAAUCAGGGUUUUGAAtt
Smad3	UGUUUUCGGGGAUGGAAUGtt	CAUUCCAUCCCCGAAAACAtt
Smad4	AAACUCAUCCUGAGUAUGCAU	GCAUACUCAGGAUGAGUUUUG
ATF4	AAUCAAACUCCUUCAAAUCdTdT	GAUUUGAAGGAGUUUGAUUdTdT
Negative control	UUCUCCGAACGUGUCACGUtt	ACGUGACACGUUCGGAGAAtt

**Table 2:** Sequences of bovine gene-specific sense and antisense primers.

Gene	Sense Primer (5′→3′)	Antisense Primer (5′→3′)
CSE	TCTCTTGGAGCAGTTCCATCTCCTA	GCAGCCCAGGATAAATAACCTTTTC
CBS	GGACTCGGTGCGGAACTACA	GGCAACACGGTCAGCGG
3-MST	GCAGTGGGTGGCTGAGGC	CGATGTCAAAGAAGGCGGC
CARS2	GAGGCGACAGGTACGGCAAG	CAGACTGGCGATGGTGGAAC
ALK1	CAACCACTACTGCTGCTACA	CCATCTCCTTGAGGCTGC
ALK5	GTCTGCTTTGTCTGTATCTCACTCA	TCCTCTTCATTTGGCACTCG
Smad2	CAGAATACCGAAGGCAGACG	TGAGCAACGCACTGAAGG
Smad3	ACTACAGCCATTCCATCC	ATCTGGTGGTCACTGGTCTC
Smad4	CTCCTATTTCTAATCATCCTGCTCC	TCTCAATGGCTTCTGTCCTGTG
ATF4	TGGTCTCAGACAACAGCAAG	AGCTCATCTGGCATGGTTTC
B2M	CCATCCAGCGTCCTCCAAAGA	TTCAATCTGGGGTGGATGGAA
